# The effect of omega-3 fatty acids on a biomarker of head trauma in NCAA football athletes: a multi-site, non-randomized study

**DOI:** 10.1186/s12970-021-00461-1

**Published:** 2021-09-27

**Authors:** Jeffery L. Heileson, Anthony J. Anzalone, Aaron F. Carbuhn, Andrew T. Askow, Jason D. Stone, Stephanie M. Turner, Lyn M. Hillyer, David W. L. Ma, Joel A. Luedke, Andrew R. Jagim, Jonathan M. Oliver

**Affiliations:** 1 Department of Health, Human Performance, and Recreation, Baylor University, Waco, TX USA; 2 Wake Forest School of Medicine, Winston-Salem, NC USA; 3 University of Kansas Medical Center, Kansas City, KS USA; 4 Nutrition and Exercise Performance Laboratory, University of Illinois at Urbana-Champaign, Champaign, IL USA; 5 Rockefeller Neuroscience Institute, West Virginia University, Morgantown, WV USA; 6 Department of Kinesiology, Texas Christian University, Fort Worth, TX USA; 7 Department of Human Health and Nutritional Sciences, University of Guelph, Guelph, Canada; 8 Athletics Department, University of Wisconsin – La Crosse, La Crosse, WI USA; 9 Sports Medicine, Mayo Clinic Health Systems, Onalaska, WI USA

**Keywords:** Docosahexaenoic Acid, American Football, Neurofilament Light, Concussion, Brain, Eicosapentaenoic Acid

## Abstract

**Background:**

American-style football (ASF) athletes are at risk for cardiovascular disease (CVD) and exhibit elevated levels of serum neurofilament light (Nf-L), a biomarker of axonal injury that is associated with repetitive head impact exposure over the course of a season of competition. Supplementation with the w-3 fatty acid (FA) docosahexaenoic acid (DHA) attenuates serum Nf-L elevations and improves aspects of CVD, such as the omega-3 index (O3I). However, the effect of combining the w-3 FA eicosapentaenoic acid (EPA) and docosapentaenoic acid (DPA) with DHA on, specifically, serum Nf-L in ASF athletes is unknown. Therefore, this study assessed the effect of supplemental w-3 FA (EPA+DPA+DHA) on serum Nf-L, plasma w-3 FAs, the O3I, and surrogate markers of inflammation over the course of a season.

**Methods:**

A multi-site, non-randomized design, utilizing two American football teams was employed. One team (*n* = 3 1) received supplementation with a highly bioavailablew-3 FA formulation (2000mg DHA, 560mg EPA, 320mg DPA, Mindset®, Struct Nutrition, Missoula, MT) during pre-season and throughout the regular season, while the second team served as the control (*n* = 35) and did not undergo supplementation. Blood was sampled at specific times throughout pre- and regular season coincident w ith changes in intensity, physical contact, and changes in the incidence and severity of head impacts. Group differences were determined via a mixed-model between-within subjects ANOVA. Effect sizes were calculated using Cohen’s *d*for all between-group differences. Significance was set *a priori *at *p*< .05.

**Results:**

Compared to the control group, ASF athletes in the treatment group experienced large increases in plasma EPA (*p* < .001, *d* = 1.71) and DHA (*p* < .001, *d* = 2.10) which contributed to increases in the O3I (*p* < .001, *d* = 2.16) and the EPA:AA ratio (*p* = .001, *d* = 0.83) and a reduction in the w-6: w-3 ratio (*p* < .001, *d* = 1.80). w-3 FA supplementation attenuated elevations in Nf-L (*p* = .024). The control group experienced a significant increase in Nf-L compared to baseline at several measurement time points (T2, T3, and T4 [*p* range < .001 – .005, *d*range = 0.59-0.85]).

**Conclusions:**

These findings suggest a cardio- and neuroprotective effect of combined EPA+DPA+DHA w-3 FA supplementation in American-style football athletes.

**Trial registration:**

This trial was registered with the ISRCTN registry (ISRCTN90306741).

## Background

Despite intense physical training, American-style football (ASF) athletes exhibit an increased risk of developing cardiovascular disease (CVD) [1]. A growing body of evidence describes relatively unfavorable cardiovascular risk factor profiles among ASF athletes [2–4]. For ASF athletes, increased body mass may lead to longer careers, more playing time, and, for those competing at the highest level, greater salaries [5]. High body mass increases the risk of obesity which is linked to CVD; however, an increased incidence of CVD in ASF athletes is not limited to those with increased body mass. A large percentage of ASF athletes, independent of body mass, have hypertension [3] and dyslipidemia [6], and sport-participation at the elite level is a stimulus for oxidative stress and inflammation [7], all contributors to CVD.

American-style football is uniquely associated with a high incidence of mild traumatic brain injury (mTBI), also known as concussion. Despite the high incidence of clinically discernible head injuries (i.e., concussions), routine exposure to repetitive subconcussive head impacts (RHI) is another potentially harmful consequence of ASF participation [8]. There is emerging evidence that RHI, in the absence of a clinically discernible injury, is linked to deleterious neurological changes [9], and a lifetime of RHI as a result of sport-participation may have long-term implications on neurological health [10, 11]. In fact, sport-related RHI, in the absence of overt functional disturbances or clinically diagnosed injuries, results in quantifiable structural [9, 10, 12, 13] and functional changes [14–16] within the brain.

The omega-3 polyunsaturated fatty acids (ω-3 FA), eicosapentaenoic acid (EPA; 20:5, ω-3) and docosahexaenoic acid (DHA; 22:6, ω-3), exhibit an array of biological functions and are vital to cardiovascular and neurological health [17]. The O3I is the sum of EPA and DHA levels in erythrocyte membranes, expressed as a percentage of total erythrocyte fatty acids [18], and is a valid marker of ω-3 FA intake and tissue status [19] and is associated with reduced CVD risk [20]. Although ω-3 FAs can be obtained through the diet, many individuals, including athletes [21], consume very low amounts, with several recent studies confirming that a large percentage of athletes have a low omega-3 index (O3I) [21, 22].

Humans [23], especially men [24], have a limited ability to synthesize EPA and DHA from the plant-based precursor ω-3 FA, alpha-linolenic acid (18:3). The most common and readily available sources of dietary EPA and DHA are fish, seafood, and supplements (from algae or fish). As such, ω-3 FA supplementation may be a viable strategy to mitigate the detrimental cardiovascular and neurological consequences experienced by many ASF athletes.

While there is speculation regarding the precise role of ω-3 FAs in CVD [25], the evidence to date suggests that EPA and DHA intake effectively reduces the incidence of certain CVD outcomes in a dose-dependent manner [26, 27]. EPA is typically recognized as the ω-3 FA component responsible for positive outcomes [28]; however, DHA is also associated with improvement in CVD risk factors, particularly those known to affect ASF athletes, namely, hypertension, hyperlipidemia, inflammation, and oxidative stress [29, 30]. Much less is known about the contribution of docosapentaenoic acid (DPA; 22:5, ω-3) to CVD risk factors, although the current evidence suggests that DPA may have similar effects as DHA [31] and EPA [32].

Emerging evidence for the role of ω-3 FAs in neuroprotective strategies is promising yet understudied. Animal models of brain injury indicate that prophylactic ω-3 FA supplementation is an effective strategy to mitigate brain damage and promote recovery [33–37]. Traumatic brain injury (TBI), including RHI, leads to a reduction in brain DHA content [38], and low DHA levels exacerbate the response to brain injury [39, 40]. While there are numerous barriers to studying RHI in humans, the quantification of blood biomarkers is an easy and cost-effective method for detecting neurological damage caused by RHI. Of the recently identified surrogate biomarkers of head trauma, serum neurofilament light (Nf-L) has been suggested to be sensitive and specific in regard to detecting neuroaxonal injury [41, 42], a characteristic of TBI [43]. Recent work by our research group indicates that the quantification of serum Nf-L may be useful for detecting the neurological damage linked to RHI sustained during a season of collegiate ASF [44, 45]. Additional work by our group demonstrated that daily supplementation with DHA exerts a neuroprotective effect in ASF athletes by attenuating elevations in serum Nf-L [46].

The ω-3 FAs EPA and DPA have independent and shared, complementary and synergistic neuro- and cardioprotective effects and might aid heart and brain health in athletes to a greater extent when combined [47]. Thus, exploring the potential cardio- and neuroprotective effects of DHA, DPA, and EPA administration was the foundation of the current study. We sought to examine the effect of supplemental ω-3 FAs on changes in a surrogate biological marker of neurological injury, serum Nf-L, and fatty acids over the course of a competitive season in ASF athletes. We hypothesized that ASF athletes supplemented with ω-3 FAs throughout the duration of their competitive season would exhibit improved cardiovascular risk markers and lower levels of serum Nf-L throughout the course of the season compared to their counterparts not supplementing with ω-3 FAs.

## Methods

### Participants

**Fig. 1:**
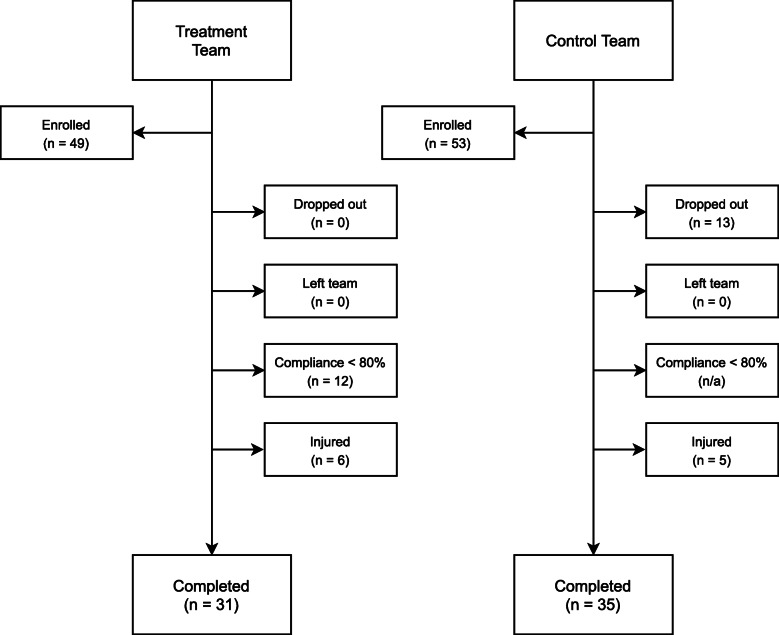
CONSORT diagram

This study was conducted according to the Declaration of Helsinki guidelines and registered with the ISRCTN registry (ISRCTN90306741). All procedures involving human subjects were approved by the Institutional Review Board of Texas Christian University (1605-053-1605). All volunteers were male National Collegiate Athletic Association (NCAA) American football athletes who were medically cleared to participate in NCAA university athletics by their respective team’s medical staff. Only those athletes who were at least 18 years of age or older were recruited and written consent was obtained from all participants. Exclusion criteria included the use of long-term anti-inflammatory therapy (≥ 20 days), the use of anti-hypertensive medications, the use of medications known to affect blood lipids, the consumption of fish oil or ω-3 FA supplementation, and self-reported consumption of more than two servings of fish per week. In addition, athletes who were injured (including, but not limited to, concussive injuries), became ill, were unable to participate in regularly scheduled conditioning, practice, or competitions, or those who did not meet the threshold compliance standards for daily supplementation (≥ 80 %) were excluded. A consort diagram is provided in Fig. 1 outlining the reasons for dropout and/or exclusion.

### Experimental design

A multi-site, non-randomized study design was utilized to examine the effect of daily supplementation with ω-3 FA on plasma fatty acids and peripheral levels of Nf-L over the course of an NCAA American football season. Two geographically distinct NCAA American football teams were recruited. The team that received treatment, (treatment team), was an NCAA Division I team; the team that did not receive treatment, control team, competed at the NCAA Division III level.

Blood was obtained from participants at various time points throughout the course of the study. Prior to the start of each team’s respective season and based on each team’s travel and competition schedule, time points were identified whereby peripheral blood samples could feasibly be obtained. The time points were also chosen to coincide with changes in practice intensity, the amount of physical contact, and the number and magnitude of head impacts that are known to be sustained by NCAA American football athletes (38, 39). Both teams followed a conventional American collegiate football schedule: several weeks of intense, full-day practices prior to the beginning of fall classes (“pre-season camp”), during which time conventional to-the-ground tackling could take place; then a period of less intense, non-tackle, afternoon-only practices before the competitive season began; then similar non-tackle practices between regular-season games usually 7 but occasionally 14 days apart.

**Fig. 2:**
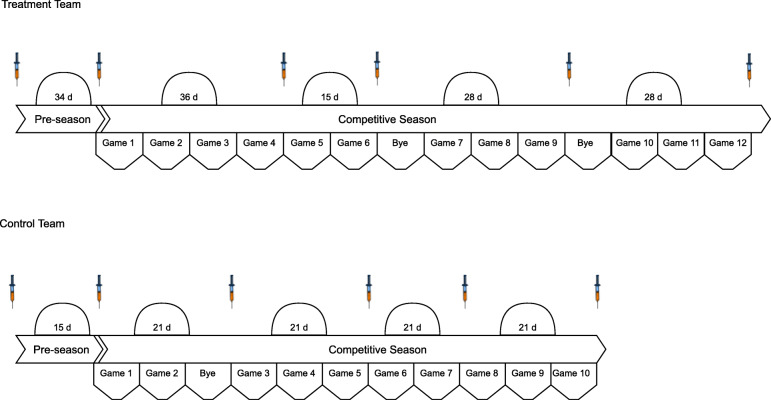
Study timeline and blood sampling schedule for each team

A baseline blood sample (T1) was obtained 7-days prior to the initiation of ω-3 FA supplementation and following a minimum 14-week period of non-contact prior to the commencement of official team practices. The second sample (T2) was obtained at the conclusion of pre-season camp (Post-Camp; T2) and before the first game of the regular season. The remaining blood samples were collected throughout the competitive season on the Monday following either a bye week or a Saturday game (48 h) (T3-T6). During the competitive season, tackles during practice were not taken to the ground, hit and wrap only. Additionally, athletes from both teams generally completed 3 strength and conditioning sessions per week. A visual depiction of the blood sampling schedule for each team a is presented in Fig. 2.

### Supplementation

On enrollment, participants from both teams were advised as to which foods were high in ω-3 FA and asked to limit servings to no more than 2 per week for the duration of the study. For the treatment team, 7-days following the initial blood sample (T1), daily supplementation began. The supplement administered was a highly bioavailable ω-3 FA formulation (Mindset®, Struct Nutrition, Missoula, MT) consisting of 2,000 mg of DHA, 560 mg of EPA, and 320 mg of DPA. Prior to commencement of the study the supplement was independently analyzed by a third party laboratory with A2LA accreditation using ISO17025 accredited screening methods for the presence of banned substances and contaminates (COA # 16,932; LGC Sciences, Inc.). Study personnel administered the supplement at least 4 times per week through the end of the regular season. Throughout the course of the study the supplement was not administered on days that the team was traveling for away games due to lack of access by study personnel as well as on days that those athletes supplementing were not required to report for official team activities. The supplement was not taken with a meal. Supplementation concluded 1 day prior to the last regular season game and 3 days prior to the final blood sample (T6). The total number of days that elapsed between the first day of supplementation and the last day of supplementation was 131 days; supplements were administered a total of 89 days. Supplement adherence and administration was conducted and monitored daily via visual supervision by research personnel. Supplement compliance and threshold for inclusion in the analysis was set at ≥ 80 % of the 89 days that supplements were administered by study personnel.

### Blood sampling and preparation

Non-fasting, supine blood samples were collected via venipuncture from the antecubital fossa region using standard phlebotomy procedures. Samples were collected in spray-coated K_2_ ethylenediaminetetraacetic acid (EDTA) vacutainer tubes and serum vacutainer tube with no additive (BD Diagnostics, Franklin Lakes, NJ, USA). All samples were centrifuged at 2,000 g for 30 min at 4^o^ Celsius (Beckman Coulter, Allegra X-15R, Brea, CA, USA) within 30 min of collection. Aliquots of serum and plasma were collected via sterile pipette from the vacutainer tubes and were immediately transferred to pre-labeled de-identified polypropylene vials for storage at -80 °C.

### Biomarker quantification

Serum Nf-L (Simoa™ Beta Kit) concentrations were measured using digital array technology on a Single Molecule Array (Simoa™) HD-1 Analyzer, software version 1.5 (Quanterix; Lexington, MA). The same lot of kits were used for each assay. Prior to analyses, samples were diluted ¼. All samples were above the lower limit of quantification (Nf-L, 0.171 pg•mL^− 1^). The lower limit of detection was 0.048 pg•mL^− 1^. Duplicates were run with a median dose coefficient of variation of 5 %.

### Plasma fatty acid composition

Plasma samples were thawed on ice. A 50 µL aliquot was removed and added to 3 mL of 2:1 chloroform: methanol (v: v). Samples were vortexed for 1 min then 550 µL of 0.1 M KCl was added to each tube with brief vortexing to mix. The tubes were spun at 1460 rpm for 10 min at 21 °C to separate phases. The lower phase was extracted and dried under nitrogen gas. Samples were methylated in 300 µL of hexane and 1 mL of 14 % boron trifluoride methanol for 1 h at 100 °C. Methylation was terminated by the addition of 1 mL dH_2_0 and 1 mL hexane. The tubes were spun at 1460 rpm for 10 min at 21 °C to separate phases. The top layer was extracted and dried under nitrogen gas. Samples were reconstituted in 400 µL of hexane for fatty acid analysis by gas chromatography.

Fatty acid methyl esters were separated on an Agilent 7890 A gas chromatograph equipped with a flame ionization detector and fused-silica capillary column (SP-2560; 100 m, 0.20 μm film thickness, 0.25 mm internal diameter). The column had a hydrogen pressure of 19.5 psi and a constant flow of 1.2 mL•min^− 1^. 1 µL of prepared sample was injected into a split (5:1) injector set at a temperature of 250 °C, a pressure of 19.5 psi and a hydrogen flow of 10.2 mL•min^− 1^. The oven was initially set at 60 °C, followed by 5 temperature ramps as described: ramp 1, increased by 13 °C•min^− 1^and held at 170 °C for 4 min; ramp 2, increased by 6.5 °C •min^− 1^ to 175 °C; ramp 3, increased by 2.6 °C •min^− 1^ to 185 °C; ramp 4, increased by 1.3 °C •min^− 1^ to 190 °C and finally ramp 5, increased by 13 °C•min^− 1^ to 240 and held for 25 min for a total run time of 49.8 min. The detector was set at 250 °C with a hydrogen flow of 30 mL•min^− 1^, air flow of 450 mL•min^− 1^ and a nitrogen flow of 10 mL•min^− 1^. Fatty acid peaks were identified by comparing their respective retention times to authentic fatty acid methyl ester standards (NuChek, Elysian, MN, USA) using Agilent OpenLab CDS EZCrome (Edition A.04.06 version 1.255.227, 2014).

To convert plasma long-chain ω-3 FA values into the equivalent erythrocyte omega-3 index, a validated conversion equation was used [48]. Given that CVD is an inflammatory condition, evaluation of the ω-6:ω-3 ratio, a marker of the balance between pro-inflammatory and anti-inflammatory mediators was calculated [49]. Finally, the EPA to arachidonic acid (EPA:AA) ratio, a reliable CVD risk marker was calculated [50].

### Statistical analysis

The distribution of all data was evaluated by the Kolmogorov-Smirnov and Shapiro-Wilk Tests. Following Levene’s test of equality of variance, baseline values were compared between groups using an independent samples t-test. The examination of the effect of ω-3 FA supplementation on Nf-L, plasma fatty acids, O3I, ω-6:ω-3, and EPA:AA were analyzed using a mixed-model between-within subject repeated measures analysis of variance (ANOVA) in SPSS V.27 (IBM Corporation; Armonk, NY). Timepoint differences were determined by separate one-way ANOVAs. Pairwise comparisons post hoc analyses were used where needed to examine within subject differences. Effect sizes were calculated using Cohen’s *d* for all between-group differences. The effect magnitudes were classified as follows: *d* < 0.20, trivial; *d* = 0.20–0.49, small; *d* = 0.50–0.79, moderate; *d* ≥ 0.80, large [51].

Given that some American football athletes are known to have a higher number of total contact hours due to the number of plays (repetitions) per game, serum Nf-L was compared across starters and non-starters. A three-way ANOVA was employed to determine if there were any group, time, starter status main or interaction effects. Starters were defined as athletes known to go out with the first or second team, first or second on the depth roster, and take a majority of the repetitions (~ 20–40 + per game). Categorization of starters resulted in nineteen on the control team (*n* = 19) and ten (*n* = 10) on the treatment team.

## Results

### ω-3 supplementation and plasma fatty acids

**Fig. 3:**
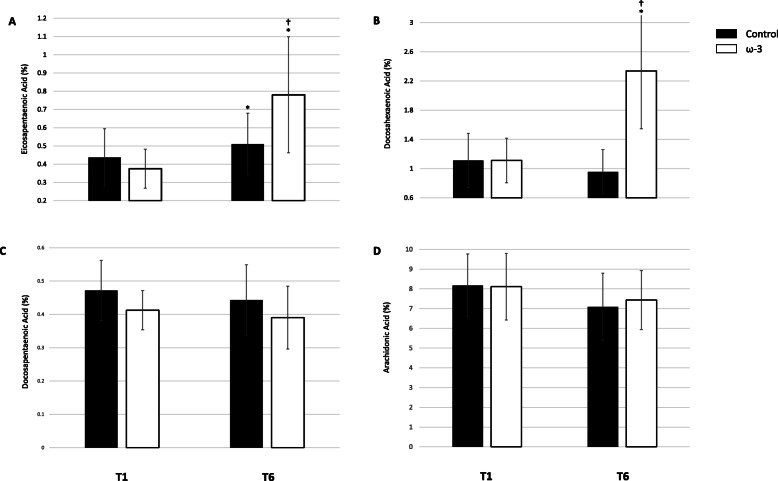
Effect of ω-3 fatty acid (FA) supplementation from baseline (T1) to the end of season (T6) on proportion (**A**) eicosapentaenoic acid (EPA), **B** docosahexaenoic acid (DHA), **C** docosapentaenoic acid (DPA), **D** arachidonic acid (AA) of total plasma fatty acids; *indicates a significant difference from baseline (*p* < .05); †indicates a significant difference between groups (*p* < .01). All data are mean ± SD

Baseline (T1) and post-season (T6) EPA, DHA, DPA, AA data based on percentage of total plasma fatty acids are presented in Fig. 3 A, B, C, and D, respectively. At T1, plasma DHA, EPA and AA were similar between groups; however, plasma DPA was higher in the control group (*p* = .049). There was a significant treatment and time effect for DHA (*p* < .001), EPA, (*p* < .001), and AA (*p* = .003). In the control group, EPA increased by 54.6 % from T1 to T6 (*p* = .012), although the magnitude was considered small (*d* = 0.45). DHA (*p* = .529) and AA (*p* = .254) did not change during the season. In the ω-3 FA supplementation group, EPA (*p* < .001) and DHA (*p* < .001) significantly increased by 110.9 % (*d* = 1.71) and 109.8 % (*d* = 2.10), respectively. Notably, DPA and AA both significantly decreased by 9.5 % (*d* = 0.56, *p* < .001) and 11.8 % (*d* = 0.50, *p* = .001), respectively. Supplement compliance over the course of the study was 93 %.

### ω-3 Supplementation, the Omega-3 Index, and the ω-6:ω-3 and EPA:AA ratio

**Fig. 4:**
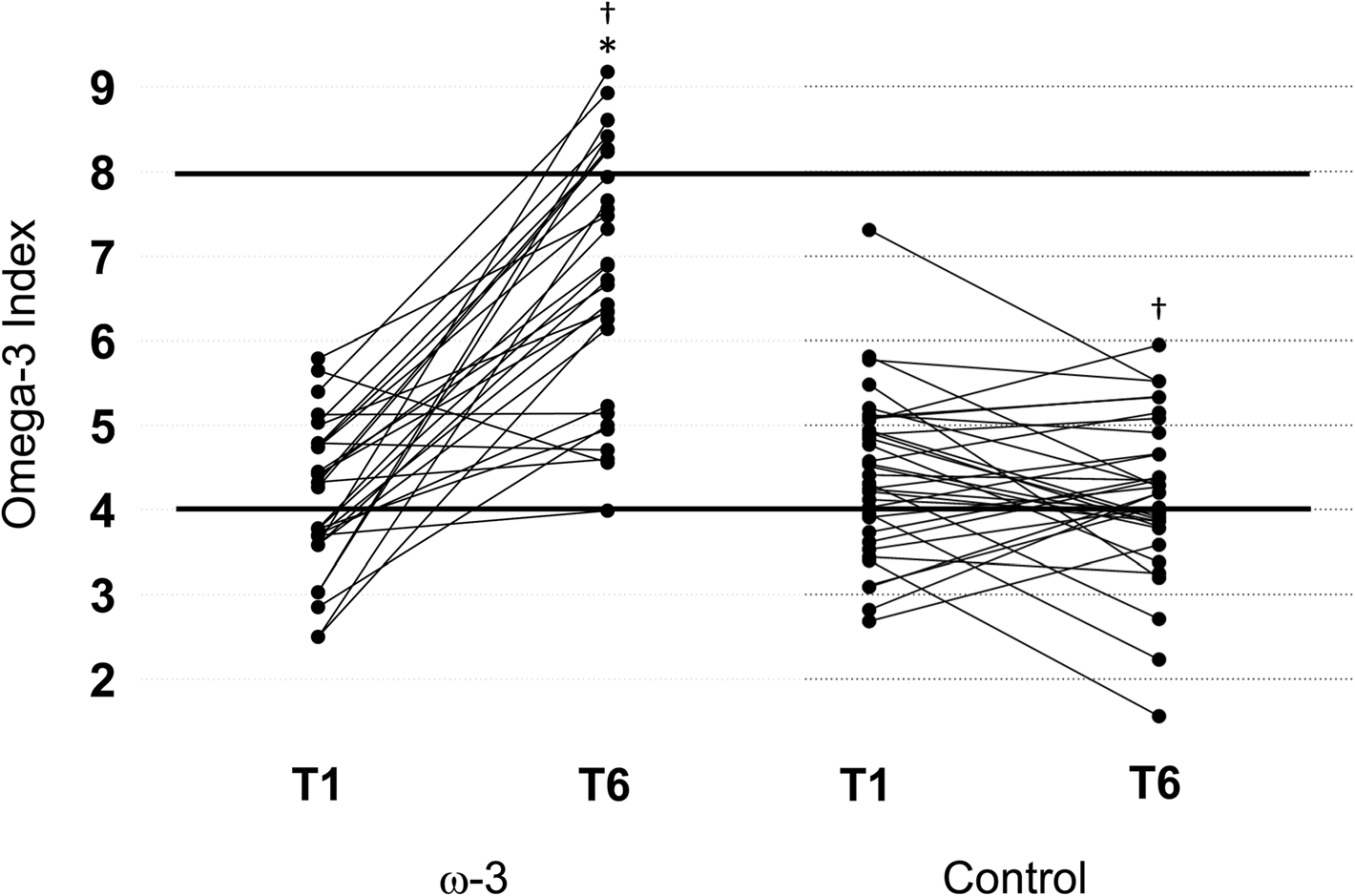
Individual changes in the Omega-3 Index (O3I) in the treatment (*n* = 30) and control (*n* = 35) groups during a NCAA football season. The solid lines indicate the cutoff values for low- and high-risk categories. Values < 4 % are high-risk and values > 8 % are low-risk. Red blood cell O3I was calculated from plasma using a validated equation [48]. *indicates a significant difference compared to baseline (*p* < .001); †indicates a significant difference between groups (*p* < .001)

Figure 4 shows the Individual O3I responses based on group. The average O3I for all athletes at baseline was 4.29 % ± 0.92 (range: 2.49–7.31). At T1, there were no differences in the calculated erythrocyte O3I between groups (*p* = .228). There was a significant group by time interaction, *F*(1, 63) = 71.914, *p* < .001. From T1 to T6, the calculated erythrocyte O3I significantly increased in the ω-3 FA group, (71.6 %, *p* < .001), indicating a very large effect (*d* = 2.16), and did not change in the control group (*p* = .116).

**Fig. 5:**
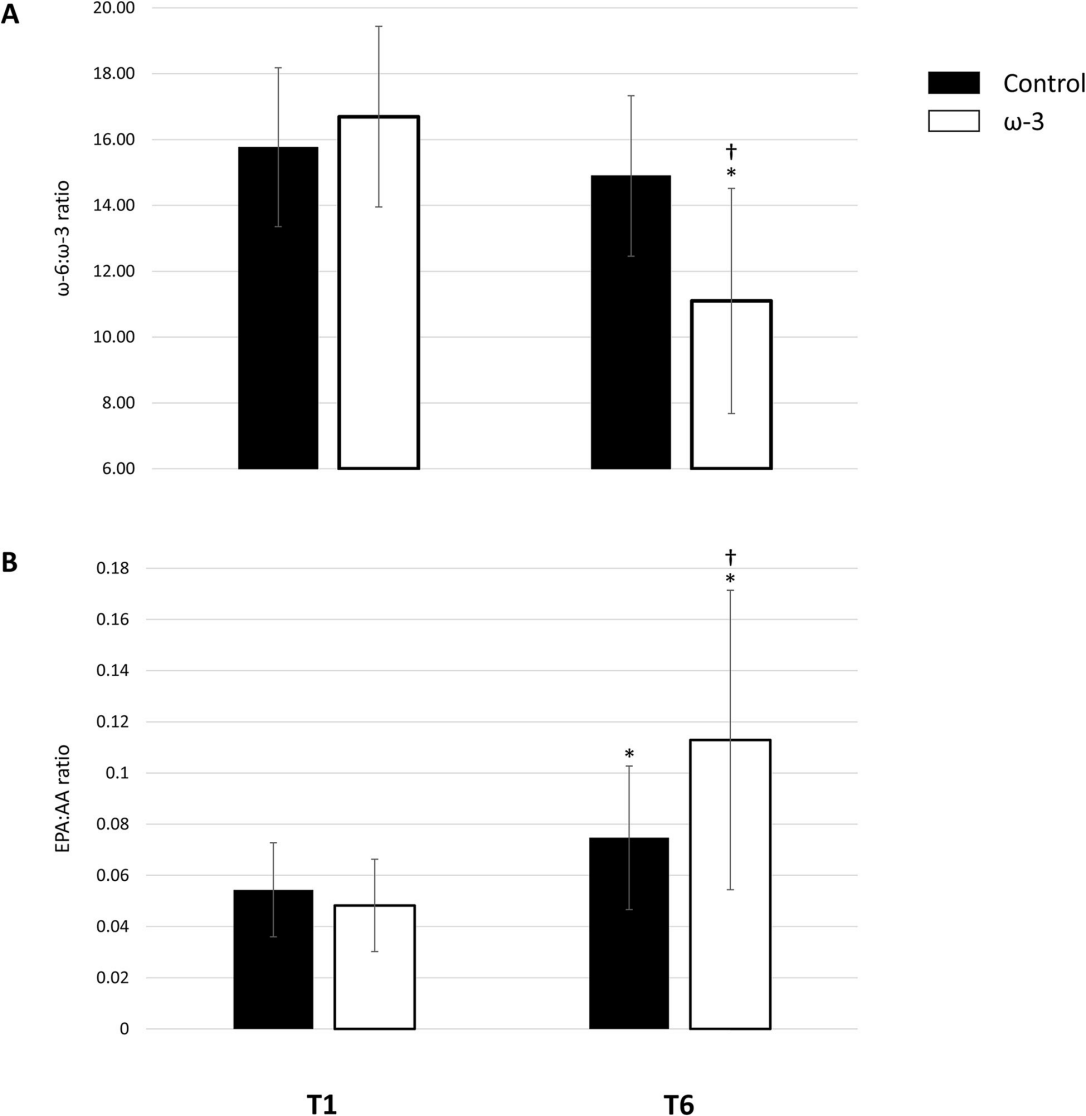
Effect of ω-3 fatty acid (FA) supplementation from baseline (T1) to the end of season (T6) on the (**A**) ω-6:ω-3 ratio, **B** eicosapentaenoic acid (EPA):arachidonic acid (AA) ratio; *indicates a significant difference from baseline (*p* < .001); †indicates a significant difference between groups (*p* < .001). All data are mean ± SD

Figure 5 A and B show data for the ω-6:ω-3 and EPA:AA ratio, respectively. For the ω-6:ω-3 ratio, there was a significant treatment and time interaction *F*(1,63) = 27.689, *p* < .001. Throughout the season, the ω-6:ω-3 ratio significantly decreased in the ω-3 FA group (32.1 %, *p* < .001), a very large effect (*d* = 1.80), and did not change in the control group (*p* = .092). Similarly, there was a group by time interaction effect for the EPA:AA ratio, *F*(1,63) = 18.059, *p* < .001. Baseline EPA:AA ratios were similar between groups (*p* = .183). Interestingly, the EPA:AA significantly increased in the control (*p* < .001) and ω-3 FA (*p* < .001) group; however, they were significantly different at the end of the season (*p* = .001), resulting in a large between group difference (*d* = 0.83). In the ω-3 FA supplementation group, the EPA:AA ratio increased by 154.9 % (*d* = 1.49); whereas the control group exhibited a 44.1 % (*d* = 0.86) increase, mediated by the aforementioned increase in EPA and insignificant change in AA status over the course of the season.

### ω-3 Supplementation and Serum Nf-L

**Fig. 6:**
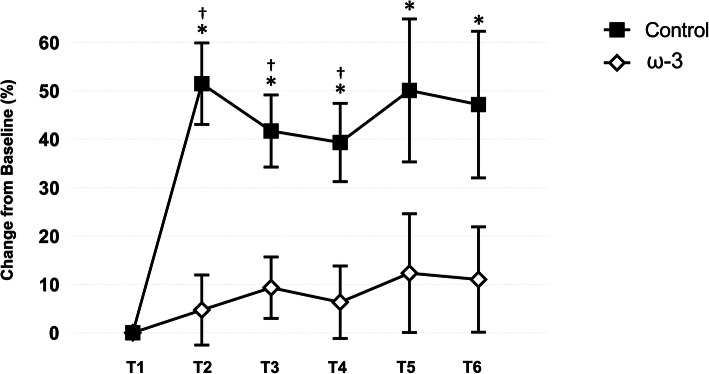
Effect of ω-3 fatty acid (FA) supplementation on serum neurofilament light (Nf-L; % change from baseline) in the control and treatment teams. *indicates a significant difference compared to baseline (*p* < .05); †indicates a significant difference between groups (*p* < .05). All data are mean (± SE)

The effect of ω-3 FA supplementation on serum Nf-L is presented in Fig. 6. Differences between the control and treatment teams in serum Nf-L prior to the start of camp and ω-3 FA supplementation were not significant (*p* = .899). Based on percent change from T1, there was a significant between group effect (*p* = .003), time effect (*p* < .001), and treatment by time effect (*p* = .024) for Nf-L. The time effect was mediated by the rise in Nf-L within the control group. From T1 to T2, the control group exhibited a significant (*p* < .001) 1.5 fold increase in Nf-L over baseline values indicative of a large effect (*d* = 0.85), whereas there was no change in the treatment group (*p* > .05). Serum Nf-L was significantly elevated compared to the ω-3 FA group at T2 (*p* < .001), T3 (*p* = .002), and T4 (*p* = .005). Notably, Nf-L remained elevated in those athletes not receiving ω-3 FA supplementation (*d* range = 0.59–0.85) throughout the competitive season, whereas the change in Nf-L in the treatment were not significant (p > .05) and only considered trivial to small (*d* range = 0.11–0.23) in magnitude.

### ω-3 Supplementation and Serum Nf-L in Athletes Categorized as Starters

**Fig. 7:**
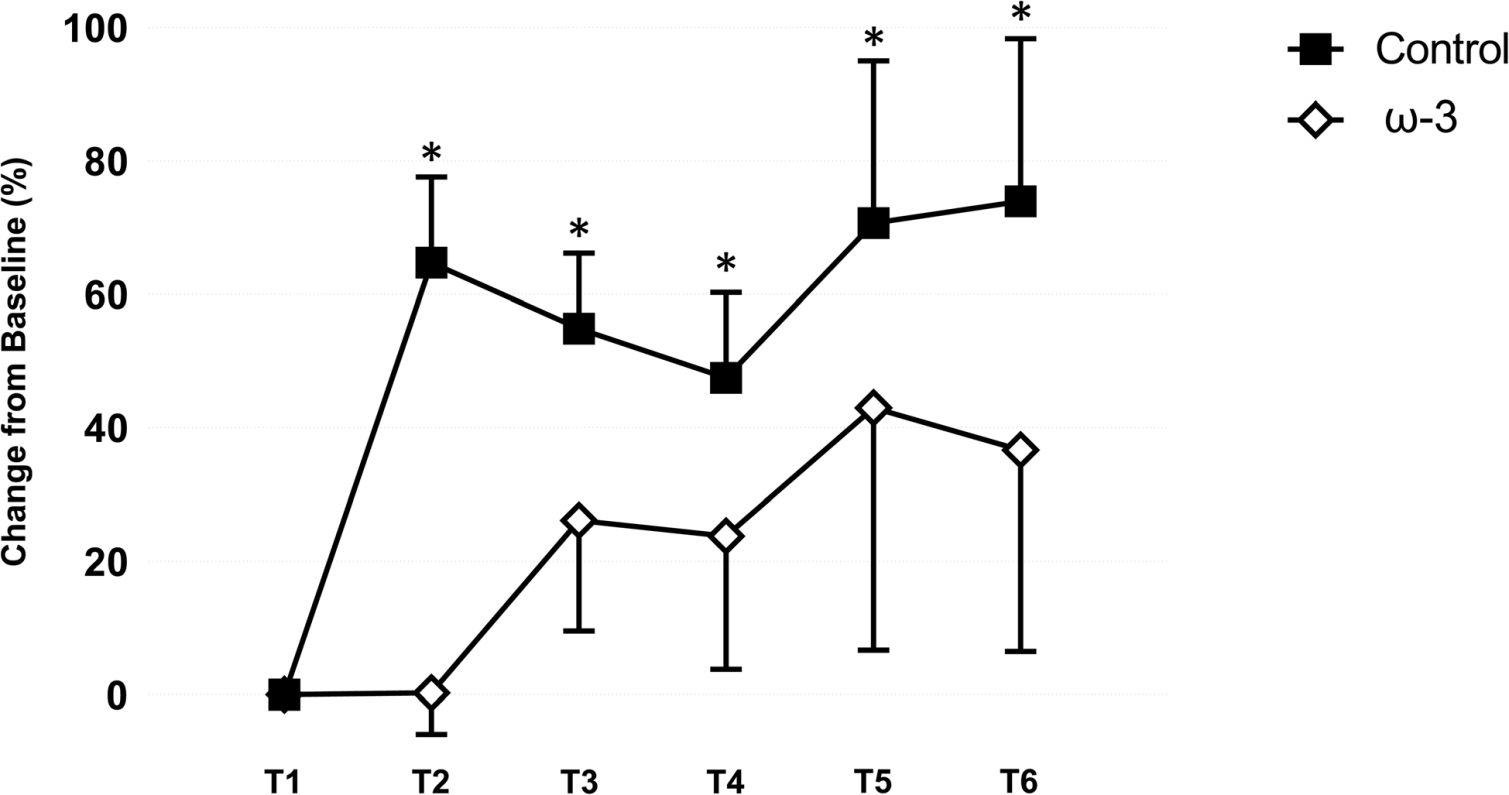
Effect of supplemental ω-3 fatty acids on serum neurofilament light (Nf-L; % change from baseline) in control and treatment teams in athletes categorized as starters. *indicates a significant difference compared to baseline (*p* < .05). All data are mean (± SE)

The effect of ω-3 FA supplementation on serum Nf-L in those athletes categorized as starters is presented in Fig. 7. As observed in all athletes, baseline values of serum Nf-L were similar between the control and treatment teams. Based on percent change from baseline, there was a significant main effect for starters (*p* = .009) and a starter by time (*p* = .003) interaction on serum Nf-L. No three-way interaction was noted between treatment group and starting status on Nf-L (*p* > .05). Interestingly, at the conclusion of camp, a large (*d* = 0.93) effect was observed in serum Nf-L for those starters on the control team, which represented a larger fold change (1.7) over baseline values than when non-starters were included. An increase was not observed in those starters receiving ω-3 FA supplementation (*d* = 0.01) post-camp (T2). Notably, the between group difference was large in magnitude (*d* = 1.05). Thereafter, serum Nf-L remained elevated in those starters on the control team (*d* range = 0.67–0.93) throughout the competitive season. When non-starters were excluded, larger mean increases were observed in those receiving ω-3 FA supplementation (*d* range = 0.01–0.52); however, those increases were, again, of lower magnitude compared to the control team starters.

## Discussion

In this study, we examined the effect of ω-3 FA (DHA, EPA, and DPA) supplementation on plasma fatty acids, cardiovascular health markers, and serum Nf-L, a surrogate marker of head trauma, in ASF athletes over the course of a competitive season. The quantity of ω-3 FA, specifically DHA (2000 mg), EPA (560 mg), and DPA (320 mg), was sufficient to increase plasma levels of DHA, and EPA, and decrease the content of AA. Notably, concomitant increases were observed in the O3I and the EPA:AA ratio, while a decrease was observed in the ω-6:ω-3 ratio. Furthermore, we demonstrated that the RHI sustained by ASF athletes result in a quantifiable increase in circulating levels of Nf-L, a marker of axonal damage. Perhaps, most importantly, we provide evidence that supplementation with DHA, EPA and DPA likely attenuates Nf-L levels associated with RHI as experienced during routine play in ASF athletes.

Recent work from our research group demonstrated that 2000 mg●day^− 1^ of supplemental DHA increased plasma DHA and reduced AA in ASF athletes; however, plasma EPA did not appreciably increase [46]. In the present study, a daily ω-3 FA supplement containing 2000 mg DHA, 560 mg EPA, and 320 mg DPA substantially increased DHA (109.8 %) and EPA (110.9 %), while reducing DPA (9.5 %) and AA (11.8 %) over the course of a competitive ASF season. Retroconversion of DHA to EPA has been observed in humans [52, 53], but a recent stable isotope study cast doubt on that notion, reporting that little to no retroconversion may actually occur [54]. Metherel and Bazinet [55] recently proposed an alternate explanation to retroconversion by speculating that DHA supplementation results in feedback inhibition of EPA metabolism resulting in an accumulation of EPA. Since DHA supplementation has been shown to decrease DPA in humans [54] and increase EPA, this is suggestive of an inhibition of EPA elongation to DPA. While additional biochemical data was not obtained, our data aligns with this updated hypothesis. In conjunction with the observed increases in plasma DHA, EPA increased (*p* < .001) and DPA decreased (*p* < .001). DPA supplementation may increase plasma and red blood cell EPA content as a recent short-term trial demonstrated that only 7 days of DPA supplementation increased concentrations of EPA with no discernable effect on plasma DHA [56]. The increase in plasma EPA observed in the current study may be attributable to the addition of EPA and DPA in our ω-3 FA supplement compared to our previous study [46]. This is an important finding, plasma and red blood cell levels of EPA and DHA attenuate CVD risk factors that are prevalent in ASF athletes such as high blood pressure, hyperlipidemia, vascular function, inflammation, and oxidative stress [30].

The O3I risk stratification system for cardiovascular health defines high risk as < 4 %, intermediate risk as 4–8 %, and low risk as > 8 % [18]. A recent study from our group found that among 404 ASF athletes, 34 % had an O3I within the high risk category while no athletes were in the category associated with low risk [22]. In a similar study conducted by Ritz et al. [21] it was reported that in a sample of 298 athletes approximately 94 % did not meet the recommended ω-3 FA intake of 500 mg DHA + EPA per day and 38 % had an O3I that put them in the high-risk CVD category. Similarly, ASF athletes in the current study had a baseline O3I of 4.29 % with values ranging from 2.49 to 7.31 %. No athlete had an O3I > 8 %, the level associated with the lowest CVD risk [18, 48]. After ω-3 FA supplementation, the mean O3I increased by 71.6 %, while the O3I fell by 3.5 % in the control group. After the season, 4 additional athletes from the control group (42.9 %) were in the high-risk category; however, ω-3 FA supplementation led to 8 athletes (26.7 %) transitioning to the low-risk category and 13 of the original 14 athletes transitioning out of the high-risk category.

Given that CVD is an inflammatory condition, measures that evaluate the balance of pro-inflammatory and anti-inflammatory mediators, such as the ω-6:ω-3 ratio, may be clinically relevant tools [49]. While the importance and usefulness of the ω-6:ω-3 ratio is continuously debated [57], the EPA:AA ratio, another evaluation of the balance between pro-inflammatory and anti-inflammatory mediators, has been proposed as a useful, simple, and reliable CVD risk marker [50]. The mechanisms underlying the health benefits of EPA and DHA on cardiovascular health have been partially attributed to EPA and DHA displacing other fatty acids, notably AA, an ω-6 FA, in phospholipids of cell membranes [58]. A decrease in cell membrane AA content and an increase in EPA and DHA alters the balance of eicosanoid and cytokine production from a generally pro-inflammatory profile to a potentially inflammation resolving profile [58]. In an earlier investigation of ASF athletes by our research group, we observed significant reductions in AA, following 2000 mg•day^− 1^ of supplemental DHA [46]. Similarly, in the current study ASF athletes in the ω-3 supplementation group exhibited significantly decreased AA (11.8 %, *p* = .005) while plasma AA increased by 8.5 % (*p* = .140) in the control group. These changes in ω-6 FA following ω-3 FA supplementation result in decreases in the ω-6:ω-3 FA ratio. High ω-6:ω-3 FA ratios, typically found in the Western diet, are alleged to promote the pathogenesis of CVD and other inflammatory diseases [59]. As expected, the ω-6:ω-3 FA ratio declined by 32.1 % (*p* < .001) with ω-3 FA supplementation. While improving the ω-6:ω-3 FA ratio, as seen in the present study, is generally considered beneficial for health, it has recently come under scrutiny as an imprecise and non-specific measure [60]. The EPA:AA ratio is a potentially much more specific and precise measure of CVD health and other inflammatory conditions [50]. Improving the EPA:AA ratio has shown stronger correlations to reducing overall CVD risk as EPA can act competitively against pro-inflammatory downstream pathways which it shares with AA [50]. In our study, ω-3 FA supplementation substantially increased the EPA:AA ratio (154.9 %), indicating a potentially favorable inflammatory profile. It is clear from the observed improvements in the O3I, ω-6:ω-3 FA, and EPA:AA ratios that the inclusion of 560 mg•day^− 1^ EPA and 320 mg•day^− 1^ DPA in a supplement containing 2000 mg•day^− 1^ DHA in ASF athletes may reduce systemic inflammation and improve CVD risk factors.

Favorable changes demonstrated by those in the treatment group of this study agree with previous research and suggest that ω-3 FA supplementation may potentially exert suppressive effects on inflammation. Thus, the changes in fatty acid profiles in this study contribute important new information specific to optimizing ω-3 FA supplementation in ASF athletes, suggesting that the combination of EPA, DPA, and DHA ω-3 FA supplementation can provide greater health benefits beyond a potential neuroprotective effect.

To date, only a few studies have documented the relationship between serum Nf-L and RHI in ASF athletes. The available evidence has demonstrated that Nf-L increases progressively throughout a season, particularly in athletes categorized as starters [44, 45]. Furthermore, Rubin et al. [61] reported that plasma Nf-L levels were influenced by the frequency and magnitude of head impact, as measured by an accelerometer-embedded mouth guard. We have previously reported that the accumulation of RHI over the course of an ASF season led to moderate to very large effect size increases in serum Nf-L levels in ASF starters [44–46]. In our current study, similar effect size increases of moderate to large were also observed in the starters of our control group.

The most novel finding of the current study was that ω-3 FA supplementation likely attenuated elevations in serum Nf-L observed over the course of a season, specifically in the treatment group categorized as starters. Previous research by Mills et al. [36] using a rodent model of TBI reported that ω-3 FA supplementation reduces markers of axonal injury. More specifically, it reduced the number of β-amyloid precursor protein-positive axons, a marker of axonal injury, in animals supplemented with ω-3 FA for 30 days before administering an experimental impact acceleration injury [36]. Our group previously reported that supplemental ω-3 FA, 2000 mg•day^− 1^ DHA, produced marked reductions in serum Nf-L compared to placebo during a competitive season [46]. However, the current study is the first to demonstrate a reduction in Nf-L following ω-3 FA supplementation containing the combination of DHA, EPA, and DPA in ASF athletes, which given the unique attributes of each FA, may provide a more substantial benefit. Further studies are warranted to examine the differential effects of those ω-3 FA.

The current study has several limitations. First, our non-treated control team competed at the NCAA Division III level while our treatment team competed at the NCAA Division I level. Therefore, given that the level of competition, size, and skill is inherently different between each group, this could potentially influence changes in serum Nf-L as well as the development of CVD risk factors. Despite these differences, the Division III control team’s serum Nf-L was also elevated, much like our previously discussed findings in Division I football athletes indicating similar increased exposure to head impacts over the course of a season [44]. Nonetheless, a comparison between similar levels of competition is suggested. Second, our study lacked monitoring of head impact data to determine the origination of Nf-L appearance in the blood. However, with a similar lack of documentation, we previously reported that nonstarter football athletes participating in strenuous practice did not have increased levels of Nf-L [45], suggesting that these changes result from head impacts, not merely physical activity. Even so, additional research using a more direct measure of head impacts (i.e., telemetry data collection) as well as the utilization of advanced imaging techniques (e.g., diffusion tensor imaging) is necessary to understand better the relationship between the number of head impacts, structural injury, and serum Nf-L levels in ASF athletes. Finally, the current study examined the effect of a combination of ω-3 FAs, which are known to have independent and complementary effects [47]. Comparing the administration of each ω-3 FA alone to the combination would provide greater insight into whether the combination augments the potential neuroprotective effect. However, a study of that magnitude is unlikely in this population.

## Conclusions

In conclusion, these findings suggest that serum Nf-L levels increase during a competitive season of ASF, starting with the end of the pre-season and that ω-3 FA supplementation, specifically the combination of EPA + DPA + DHA, has potential cardiovascular and neuroprotective effects. Our findings provide crucial information on ω-3 FA supplementation and a surrogate biological marker of head trauma in a population known to sustain RHI. Similar elevations of Nf-L have been reported following RHI in other contact sport athletes [62, 63]. These data suggest that those other contact sport athletes may also benefit from ω-3 FA supplementation, primarily since most athletes do not obtain enough dietaryω-3 FA and have relatively low levels of ω-3 FA [21, 22]. Furthermore, the notable CVD risk factors present in ASF athletes, especially linemen, can be potentially ameliorated by ω-3 FA supplementation as measured by the combination of O3I, ω-6:ω-3 FA, and EPA:AA ratios. Given the associated health functions and benefits of ω-3 FAs related to both athlete performance and well-being [46, 64–66], and that supplementation is well tolerated and safe at any age, further research is warranted to explore the potential cardiovascular, neuroprotection, and, even, musculoskeletal benefits in athletes.

## Data Availability

The data collected and analyzed during the course of this study are available from the corresponding author upon reasonable request.
